# Scutellarin Ameliorates Renal Injury via Increasing CCN1 Expression and Suppressing NLRP3 Inflammasome Activation in Hyperuricemic Mice

**DOI:** 10.3389/fphar.2020.584942

**Published:** 2020-10-22

**Authors:** Guozheng Li, Chen Guan, Lingyu Xu, Lin Wang, Chengyu Yang, Long Zhao, Bin Zhou, Congjuan Luo, Hong Luan, Wei Jiang, Chenyu Li, Yan Xu

**Affiliations:** ^1^ The Affiliated Hospital of Qingdao University, Qingdao, China; ^2^ Medizinische Klinik und Poliklinik IV, Klinikum der Universität, LMU München, München, German

**Keywords:** scutellarin, hyperuricemia, hyperuricemic nephropathy, CCN1, NLRP3 inflammasome

## Abstract

Considerable evidences have indicated that elevated uric acid (UA) was involved in renal tubular injury leading to hyperuricemic nephropathy (HN). Scutellarin is a biologically active flavonoid derived from the Chinese traditional herb *Erigeron breviscapus* Hand-Mazz, which has been widely used in the treatment of cardiovascular and cerebrovascular diseases. In the present study, we analyzed the effect of scutellarin on HN, by using C57BL/6 mice and human renal tubular epithelial cell line HK-2 which was subjected to adenine/potassium oxonate and UA to mimic a HN injury. The HN mice showed a significant decrease in renal function with the increased SCr and blood urea nitrogen (BUN) (*p* < 0.05). Hematoxylin–eosin staining results showed a histological injury in HN mice kidney tissues with severe tubular damage. Scutellarin dose dependently alleviated the renal injury of the HN model (*p* < 0.05), and a dose of 20 mg/kg/day remarkably reduced the Scr level (26.10 ± 3.23 μmol/ml vs. 48.39 ± 7.51 μmol/ml, *p* < 0.05) and BUN (151.12 ± 30.24 mmol/L vs. 210.43 ± 45.67 mmol/L, *p* < 0.05) compared with the HN model group. Similarly, scutellarin decreased NGAL, Kim-1, cystatin C, and IL-18 protein expression levels in HN mouse (*p* < 0.05). Overexpressed CCN1 could not induce NLRP3 inflammasome activation, with no change of mRNA and protein expression levels of NLRP3, ASC, and pro-caspase-1 compared with the control HK-2. However, HK-2 showed a significant NLRP3 inflammasome activation and apoptosis. Importantly, knockdown of CCN1 not only aggravated NLRP3 inflammasome activation and apoptosis but also abrogated the protective effect of scutellarin in UA-induced HK-2 injury. Thus, scutellarin might alleviate HN progression *via* a mechanism involved in CCN1 regulation on NLRP3 inflammasome activation.

## Introduction

Hyperuricemia (HUA), a common metabolic disorder of purine, is characterized by high serum uric acid (SUA)-precipitated urate crystals in both the kidneys and joints. Elevated SUA may cause the formation of monosodium urate crystals in connective tissues, eventually leading to gout. Based on the National Health and Nutrition Examination Survey from 2007 to 2008, 21.1% adult men and 21.6% adult women suffered from HUA in the United States (Zhu et al., 2012). In China, the prevalence of HUA was estimated to be 19.4% in men and 7.9% in women (Liu et al., 2015), approximately 10% of which developed into gout. Recent studies showed that HUA may have contributed to various complications including diabetes, hyperlipidemia, and hypertension (Merriman and Dalbeth, 2011; Li et al., 2013; Bonakdaran and Kharaqani, 2014), as well as an independent risk element for chronic kidney disease (CKD) (Obermayr et al., 2008).

Numerous studies revealed that elevated uric acid (UA) was associated with renal tubular injury and subsequent tubulointerstitial fibrosis, thus causing hyperuricemic nephropathy (HN), which can eventually cause CKD. Nucleotide-binding oligomerization domain-like receptor protein 3 (NLRP3) inflammasome is a multi-protein complex composed of NLRP3, and apoptotic speck protein including a caspase recruitment domain (ASC) and pro-caspase-1 (Huai et al., 2014). Pathogen-associated molecular patterns (PAMPs) and damage-associated molecular patterns (DAMPs) can be identified by NLRP3 receptor proteins, which existed in a variety of components of the body (Martinon et al., 2006). Current studies revealed that activation of NLRP3 inflammasome can be triggered by soluble UA and UA crystals, thus causing maturation of pro-inflammatory cytokines like IL-1β and leading to congenital immune defense against danger signals such as infection and metabolic disorder (Isaka et al., 2016).

Scutellarin (4′,5,6-trihydroxyflavone-7-O-glucuronide) is a biologically active flavonoid derived from the Chinese traditional herb *Erigeron breviscapus* Hand-Mazz, which has been widely used in the treatment of cardiovascular and cerebrovascular diseases (Wang Z. et al., 2016; Chledzik et al., 2018). Recent pharmacological studies have reported anti-inflammatory, antioxidant, neuroprotective, and antitumor activities (Du et al., 2015; Baluchnejadmojarad et al., 2018; Sun et al., 2018) of scutellarin. Particularly, researchers confirmed the protective effects of scutellarin against hypoxic–ischemic brain and cardiomyocyte injury mediated by antioxidant capacity (Guo et al., 2011; Wang Z. et al., 2016). Du et al. (2015) demonstrated that scutellarin protected cerebral vascular endothelial cells through activating the endothelial cGMP-activated protein kinase G pathway. Additionally, scutellarin can inhibit NLRP3-related inflammasome stimulated by ATP or nigericin in lipopolysaccharide-primed macrophages. However, so far, whether scutellarin protects renal function and reduces serum UA of HN mice remains elusive.

CCN1 as a cysteine-rich secretory protein promotes embryonic development, adhesion, chemotaxis, cell proliferation, and neovascularization (Lau, 2016), and is increased in inflammation, apoptosis, and other injury conditions (Lai et al., 2013). Our previous studies found that CCN1 showed anti-apoptosis effect in ischemic renal tubular epithelial cells (Xu et al., 2014; Li et al., 2019). In this study, we analyzed the effect of scutellarin on HN by using C57BL/6 mice and human renal tubular epithelial cell line HK-2 subjected to adenine/potassium oxonate and UA to mimic a HN injury. Further, to study the possible underlying mechanisms, the relationship between scutellarin and CCN1, the regulatory effects of CCN1 on NLRP3 inflammasome, and apoptosis in HN were detected. This study will offer an improved understanding of scutellarin, NLRP3 inflammasome, and apoptosis and CCN1, and may promote the development of strategies for HN treatment.

## Materials and Methods

### Scutellarin

Scutellarin was purchased from MCE company (CAS: 27740-01-8, purity > 98.0%, cat: HY-N0751, MedChemExpress, NJ, United States). We prepared a DMSO (cat: D8370, Solarbio, Shanghai, China) stock liquid and then added 0.9% saline to a final concentration of 8.3 g/L (18.03 mmol/L). The molecular formula of scutellarin is C_21_H_18_O_12_, with molecular weight 462.36. Before being added to the culture medium for the *in vitro* assays, the scutellarin solution was first filtered using 0.2 μm filtration membranes, and the filtration was done to sterilize the solution. The final concentration of DMSO was 0.5% in all the solutions added to the cells in this study.

### Cell Culture and Transfection

HK-2 cells, which were purchased from the Cell Bank of the Chinese Academy of Sciences (Shanghai, China), were cultivated in DMEM (HyClone, Logan, UT) containing 10% fetal calf serum (Gibco, Langley, OK) supplemented with 1% penicillin–streptomycin (Gibco), and cells were maintained in a humidified atmosphere at 37 °C with 5% CO_2_. Further, various concentrations of UA (0, 4, 8, or 16 mg/dl) for 24 h or 8 mg/dl UA for different time (24, 48, and 72 h) were performed for HK-2 cell administration. HK-2 cells were seeded at a density of 5 × 10^5^ cells/well in six-well plates for adherence. A CCN1 expression vector, which was confirmed by sequencing, was constructed by sub-cloning the full-length wild-type CCN1 coding sequence into pcDNA3.1(+) (Sangon Biotech, Shanghai, China). The empty constructed pcDNA3.1 was transfected as a control. The siRNA sequences of siRNA-CCN1 were purchased from GeneChem (Shanghai, China). Lipofectamine 3000 reagent (Invitrogen, Carlsbad, CA) was used for transfections following the instructions from the manufacturer. Stable transfectants were chosen with G418 (Life Technologies, Grand Island, NY).

### Cell Viability Assay

Cell Counting Kit-8 (CCK-8, Dojindo Molecular Technologies, Kumamoto, Japan) was used for cell viability assessment. Briefly, HK-2 cells or the transfected HK-2 cells were seeded at a density of 5 × 10^3^ cells/well in 96-well plates. 20 μL CCK-8 was added into each well and incubated for 2 h at 37 °C after UA stimulation. Further, a microplate reader (Bio-Rad, Hercules, CA) was used for the measurement of absorbance at 450 nm.

### Annexin V Apoptosis Assay

Flow cytometry was used to evaluate the number of apoptotic cells with Annexin V-FITC/PI apoptosis kit (BioVision, Milpitas, CA) according to the manufacturer’s instructions. Briefly, the HK-2 cells were seeded into six-well plates at a density of 5 × 10^5^ cells/well. Then, cells were washed with PBS twice and resuspended in 200 μL binding buffer. After adding 5 μL Annexin V conjugate and incubation for 10 min, the samples were resuspended with 200 μL binding buffer and 5 μL propidium iodide (PI). The samples were then incubated at room temperature for 30 min in the dark, and a flow cytometer (BD Biosciences, San Jose, CA) was used for distinguishing the apoptotic cells (Annexin V-FITC positive and PI-negative).

### Animals and Groups

Male C57BL/6 mice aged 8–10 weeks weighing 25–27 g were purchased from Vital River experimental animal technology company (Beijing, China) and were housed in a temperature-controlled room (23 ± 2 °C) under a 12-h light/dark cycle supplemented with food and water ad libitum. After being fed for preadaptation to the environment for a week, 36 male mice were randomly divided into six groups (six per group): control, HN mice model, scutellarin (Scu) 20 mg, HN + Scu 5 mg, HN + Scu 10 mg, and HN + Scu 20 mg. Oral administration with adenine (160 mg/kg) and potassium oxonate (240 mg/kg) mixture daily consistently for 3 weeks to establish HN mice was followed. Thereafter, scutellarin were given every day by intraperitoneal injection to evaluate the efficacy of scutellarin in HN mice. After 3-week feeding, the animals were euthanized, and the kidneys were collected for histologic examination and protein analysis. The serum samples were taken for the measurement of SUA, creatinine, and blood urea nitrogen (BUN).

All animal experiments were reviewed and approved by the Medical Ethics Committee for experimental research at the Affiliated Hospital of Qingdao University.

### Sample Collection

Mice body weights and SUA levels were noted before and after OA induction at 2-week intervals. Blood samples, which were collected from the tail vein, were left to clot for 1 h at room temperature in priced tubes, and then centrifuged at 3,000 g at 4 °C for 15 min to get the serum, which was transferred to a clean tube and immediately stored at −20°C for further detection. During drug administration, urine samples were collected using metabolic cages at 2-week intervals and then centrifuged at 3,000 g for 5 min to remove impurities. All samples were stored at −20 °C for the following assays. Specific commercial kits purchased from Nanjing Jiancheng Bioengineering Institute were used for the detection of SUA, Scr, and BUN levels according to the manufacturer’s instructions (Nanjing, China). No food was allowed 8 h before the surgery but drinking water at liberty. Mice were anesthetized using pentobarbital sodium (30 mg/kg i.p.), and blood samples were collected *via* inferior vena cava. The kidney was quickly dissected, and partial cortex tissues of the left kidney were frozen in liquid nitrogen immediately. Total protein and RNA were stored at −80 °C until being assayed after extraction from kidney tissues.

### Hematoxylin–Eosin Staining

Kidneys from all treated groups were fixed in 10% buffered formalin overnight at 4 °C and embedded in paraffin. Paraffin-embedded sections (4 μm) were deparaffinized with xylene, dexylened in successive concentrations of ethanol, and stained with hematoxylin and eosin. Tissue sections (five sections per kidney) were blindly labeled, of which 10 fields per section in total were chosen randomly and were observed by two renal pathologists.

Renal injury was graded according to the percentage of damaged tubules in the sample as previous described. In brief, 0 refers to no identifiable injury; 1 refers to mitosis and necrosis of individual cells; 2 represents necrosis of all cells in adjacent proximal convoluted tubules, with survival of surrounding tubules; 3 signifies necrosis confined to the distal third of the proximal convoluted tubules, with a band of necrosis extending across the inner cortex; and 4 means necrosis affecting all three segments of the proximal convoluted tubule. A score of 1 or 2 represents mild injury, and a score of 3 or 4 represents moderate or severe injury, respectively, in which injury included inflammatory cell infiltration, dilation of renal tubules, and interstitial edema.

### Quantitative Real-Time Polymerase Chain Reaction

RNAiso Plus reagent was used for Total RNA of the kidney tissue isolation. 500 ng RNA from each sample was reversely transcribed to cDNA using a PrimeScript^™^ RT reagent kit (Takara, Otsu, Japan), and real-time PCR was performed *via* the SYBR Green (Takara, Otsu, Japan) method with the following conditions: at 95 °C for 1 min followed by 40 cycles at 95 °C for 15 sec, 60 °C for 15 sec, and 72 °C for 45 sec, and the specificity of the PCR products was confirmed by melting curve analysis and sequencing. Primers used in this experiment are shown in Table 1. GAPDH was referred as the reference gene to normalize the mRNA quantity, and 2^−ΔΔCT^ method was used to calculate the relative expression of mRNA.

**TABLE 1:** Primers used in the article.

Gene	Forward	Reverse
h-NLRP3	GAT​CTT​CGC​TGC​GAT​CAA​CAG	CGT​GCA​TTA​TCT​GAA​CCC​CAC
m-NLRP3	ATT​ACC​CGC​CCG​AGA​AAG​G	CAT​GAG​TGT​GGC​TAG​ATC​CAA​G
h-ASC	GCC​GAG​GAG​CTC​AAG​AAC​T	AGC​TTG​TCG​GTG​AGG​TCC​AA
m-ASC	AGA​CCA​CCA​GCC​AAG​ACA​AG	CTC​CAG​GTC​CAT​CAC​CAA​GT
h-pro-IL-1β	GCT​TGG​TGA​TGT​CTG​GTC​CA	TCA​ACA​CGC​AGG​ACA​GGT​AC
m-pro-IL-1β	GCT​TGG​TGA​TGT​CTG​GTC​CA	TCA​ACA​CGC​AGG​ACA​GGT​AC
h-CCN1	TCG​GCA​GCC​TGA​AAA​AGG​GCA	TCG​CAG​CGG​AAG​CGC​ATC​TT
m-CCN1	ACC​CGG​ATT​TGT​GAG​GTG​C	GCA​GGA​ACC​GCA​GTA​CTT​GG
h-BAX	CCC​GAG​AGG​TCT​TTT​TCC​GAG	CCA​GCC​CAT​GAT​GGT​TCT​GAT
m-BAX	AGA​CAG​GGG​CCT​TTT​TGC​TAC	AAT​TCG​CCG​GAG​ACA​CTC​G
h-Bcl-2	ATC​GCC​CTG​TGG​ATG​ACT​GAG	CAG​CCA​GGA​GAA​ATC​AAA​CAG​AGG
m-Bcl-2	GGG​GCT​ACG​AGT​GGG​ATG​C	GCG​GTA​GCG​GCG​GGA​GAA​GT
h-caspase-3	TGC​ATA​CTC​CAC​AGC​ACC​TGG​TTA	CAT​GGC​ACA​AAG​CGA​CTG​GAT​GAA
m-caspase-3	CGA​TTA​TGC​AGC​AGC​CTC​AA	AGG​AGA​TGC​CAC​CTC​TCC​TT
h-caspase-9	GGT​CAC​GGC​TTT​GAT​GGA​GAT	CCA​CCT​CAA​AGC​CAT​GGT​CTT
m-caspase-9	GCT​CTT​CCT​TTG​TTC​ATC​TCC	CAT​CTG​GCT​CGG​GGT​TAC​TGC
h-GAPDH	GGA​GCG​AGA​TCC​CTC​CAA​AAT	GGC​TGT​TGT​CAT​ACT​TCT​CAT​GG
m-GAPDH	TGA​CCT​CAA​CTA​CAT​GGT​CTA​CA	CTT​CCC​ATT​CTC​GGC​CTT​G

### Western Blotting

Serum, urine, and kidney tissues were kept at sacrificed for protein extraction, and RIPA buffer was used for the sample suspension. The homogenate was centrifuged at 10,000 g for 30 min at 4 °C. 50 μg protein in mixture solution were separated with 10% SDS-PAGE and then transferred onto PVDF membranes of 0.45 μm (Millipore, Germany) incubated with 5% skimmed milk in phosphate-buffered saline at room temperature for 1 h (Li et al., 2019). After incubation with primary antibodies against and β-actin (Cell Signaling Technology, MA, United States) overnight at 4 °C, the membrane was incubated with secondary antibodies after washing with PBS and Tween 20. The target bands were detected with chemiluminescence. The antibodies against CCN1, NLRP3, ASC, pro-caspase-1, IL-1β, Kim-1, IL-18, NGAL, cystatin C, Bax, Bcl-2, and caspase-3 and caspase-9 were purchased from Abcam (Cambridge, MA, United States). All other chemicals were of analytical reagent grade.

### Statistical Analysis

All data were expressed as means ± SEM using SPSS from three repeated experiments. One-way analysis of variance was used among diverse groups, and independent samples were analyzed by Student’s *t*-test when appropriate. A value of *p* < 0.05 was regarded as statistically significant.

## Results

### Scutellarin Reduced Serum Uric Acid and Protected Renal Function of Hyperuricemic Nephropathy Mice

**FIGURE 1:**
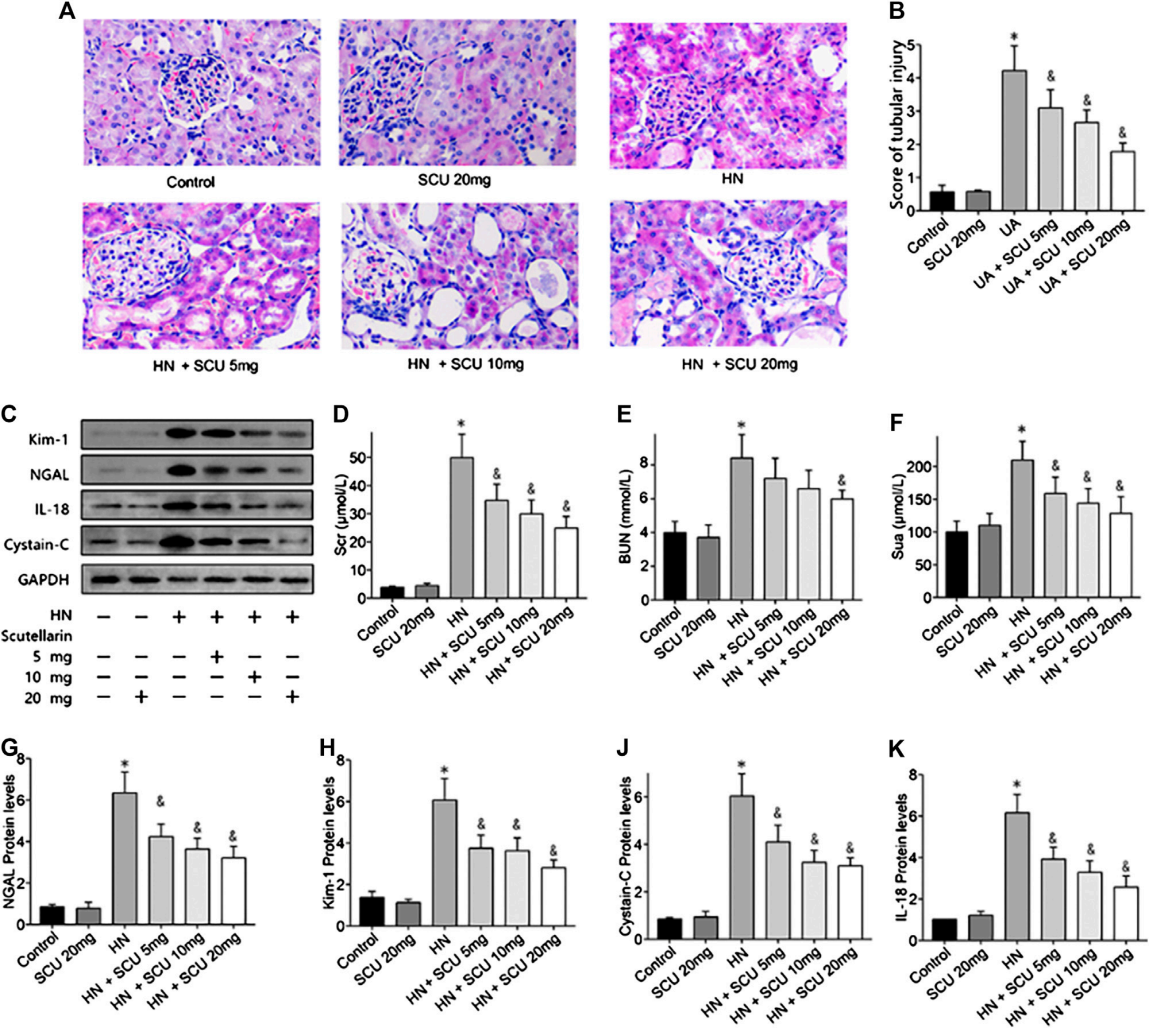
Effects of scutellarin on HN mice. Mice were with a mixture of adenine/potassium oxonate for 3 weeks to induce the HN mouse model. HE stained analysis showed scutellarin alleviate tubular damage of HN mouse (×400) **(A)** with a significant decline kidney tubular score **(B)**. Western blot showed scutellarin dose dependently decreased Scr **(D)**, BUN **(E)**, Sua **(F)**, and NGAL, Kim-1, cystatin C, and IL-18 protein expression levels in HN mouse **(C, G–K)**. *p < 0.05 vs. the control group; ^&^ *p* < 0.05 vs. HN group.

Mice were fed with adenine/potassium oxonate mixture for 3 weeks according to former studies to establish the HN mice model, and the SUA level of HN mice was 220.12 ± 14.29 μmol/L in the end (Figure 1F), which was significantly increased compared with that of the control group (165.91 ± 12.31 μmol/L; *p* < 0.05), indicating that HN mice model was successfully induced. Hematoxylin–eosin (HE) staining results revealed a severe renal tubular damage in HN mice, which was mitigated after scutellarin administration with a significant decline in kidney tubular injury score (*p* < 0.05, Figures 1A,B). Further, to study the effects of scutellarin on the HN mice, we pretreated HN mice with a different dose of scutellarin (5, 10, and 20 mg/kg/day); as a result, renal function of HN mice significantly decreased with the increased Scr and BUN, while scutellarin can alleviate the renal injury of the HN model in a dose-dependent manner (*p* < 0.05, Figures 1D,E), and a dose of 20 mg/kg/day remarkably reduced Scr (26.10 ± 3.23 μmol/ml vs. 48.39 ± 7.51 μmol/ml, *p* < 0.05) and BUN levels (151.12 ± 30.24 mmol/L vs. 210.43 ± 45.67 mmol/L, *p* < 0.05) compared with the HN group. Moreover, expressions of biomarkers of renal injury including NGAL, Kim-1, cystatin C, and IL-18 were increased while both mRNA and protein expression levels were significantly decreased after scutellarin treatment in HN mice (Figures 1C,G–K, *p* < 0.05). Importantly, scutellarin has no negative effects on renal function in normal mice when administrated with the maximum dosage that was 20 mg/kg/day (Figures 1A–K). Taken together, scutellarin could improve renal function and alleviate glomerular and tubular damage in mice with HN.

### Protective Effect of Scutellarin Is Associated with NLRP3 and CCN1

**FIGURE 2:**
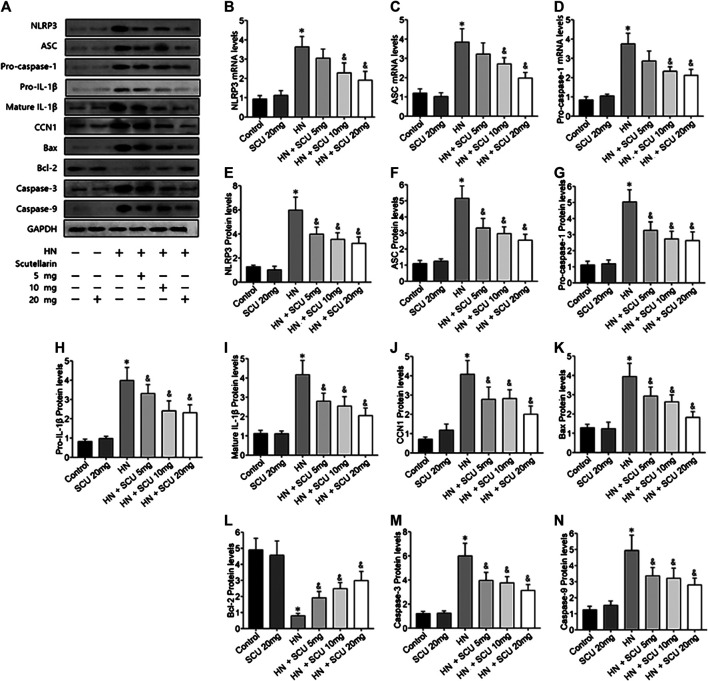
Scutellarin protective effect is associated with NLRP3 and CCN1. Western blot showed scutellarin pretreatment HN mice showed a significant dose-dependent inhibition of NLRP3 inflammasome components **(A–G)** and apoptosis-related protein **(H–N)**. The expression of CCN1 protein in HN kidney issues was unregulated and downregulated when administrated with Scu **(J)**. *p < 0.05 vs. the control group; ^&^ *p* < 0.05 vs. HN group.

Multiple studies have shown that the activation of NLRP3 inflammasome was involved in HN, which was confirmed in our study as well. The mRNA and protein expression levels of the components of the NLRP3 inflammasome including NLRP3, ASC, and pro-caspase-1 were upregulated in HN kidneys issues compared with that of the control group (Figures 2A–G). Pro–IL-1β and mature IL-1β protein contents in the kidneys were also increased in HN (Figures 2H,I). Moreover, expressions of apoptosis-related proteins, that is, Bax, cleaved caspase-3, and cleaved caspase-9, were upregulated, while Bcl-2 was downregulated in the HN kidney tissues (Figures 2K–N). Scutellarin of pretreatment HN mice showed significant dose-dependent inhibition of NLRP3 inflammasome and apoptosis, suggesting that its protective effect was associated with the NLRP3 and apoptosis. More importantly, we found that the expression of CCN1 protein, an early biomarker of apoptosis in acute kidney disease, was upregulated and downregulated when administrated with scutellarin, indicating that CCN1 was also involved in scutellarin protection of apoptosis of HN (Figure 2J).

### Effects of Uric Acid and Scutellarin in HK-2 Cells

**FIGURE 3:**
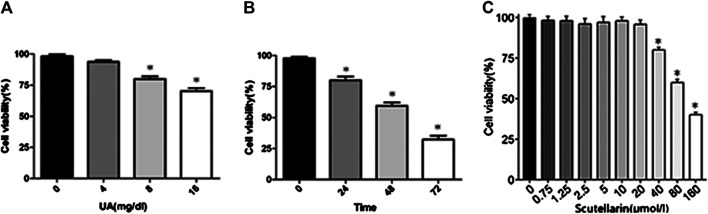
Effects of UA and scutellarin in HK-2 cells. Cell Counting Kit-8 showed UA damaged HK-2 cells in a dose- and time-dependent **(A-B)** manner, and the optimal dosage of scutellarin **(C)**. *p < 0.05 vs. the control group.

HK-2 cells were subjected to different concentrations of UA (0, 4, 8, or 16 mg/dl) for 24 h and then subjected to a CCK8 assay to detect the effects of UA on cell viability. As indicated in Figure 3A, UA inhibited HK-2 cell viability in a dose-dependent manner, particularly 8 mg/dl treated for 24 h significantly decreased cell viability (*p* < 0.05). Meanwhile, cells pretreated with 8 mg/dl UA for various times (0, 24, 48, and 72 h) showed a significant decrease of cell viability after 24 h of UA stimulation (*p* < 0.05, Figure 3B), suggesting that UA damaged HK-2 cells in a dose- and time-dependent manner. Furthermore, we studied the effects of scutellarin on cell viability. The results showed HK-2 cell viability decreased in the presence of 40 μmol/L scutellarin for 24 h (Figure 3C; *p* < 0.05); therefore, 8 mg/dl UA for 24 h and 40 μmol/L scutellarin for 24 h were selected for subsequent experiments.

### Overexpressed CCN1 Improved Uric Acid–Induced HK-2 Injury *via* Negative Regulation of NLRP3

**FIGURE 4:**
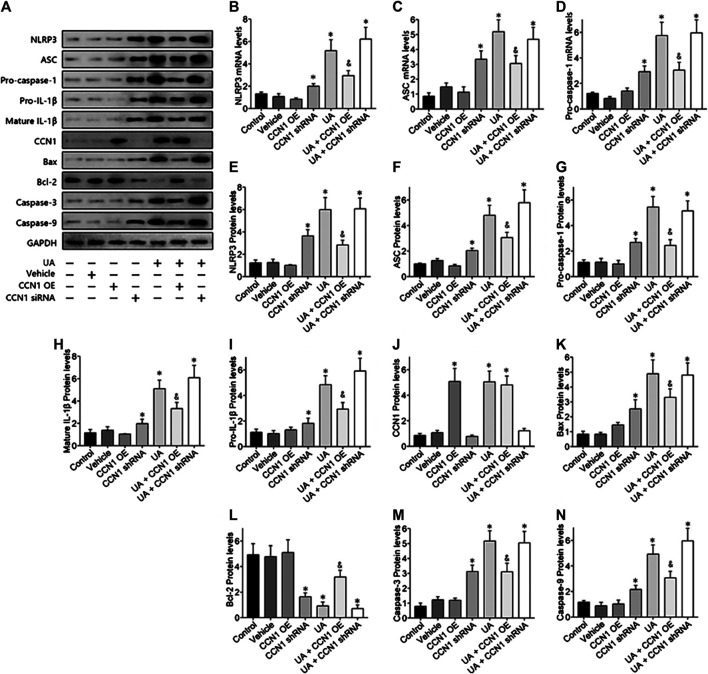
Over-expressed CCN1 improved UA-induced HK-2 injury. Western blot showed the overexpressed CCN1 significant inhibited NLRP3 inflammasome components **(A–G)** and apoptosis-related protein **(H–N)**. However, HK-2 shows a significant NLRP3 inflammasome activation and apoptosis, when knockdown of CCN1 with shRNA. *p < 0.05 vs. the control group; ^&^ *p* < 0.05 vs. HN group.

**FIGURE 5:**
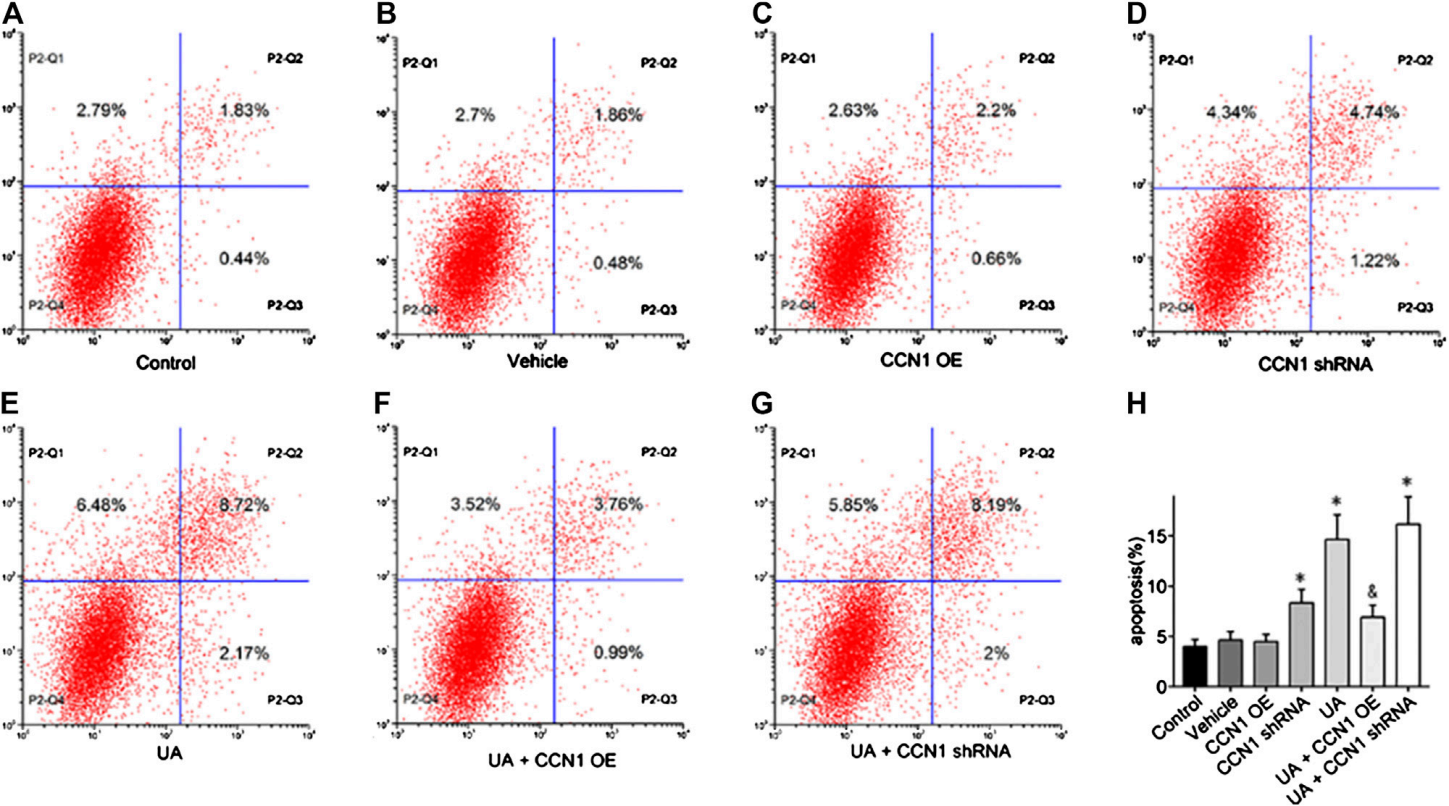
Effects of CCN1 on HK-2 cell apoptosis. Flow cytometric dot plots for HK-2 cell treated with a different manner **(A–G)**.Quantification of flow cytometry provided apoptotic rates **(H)**. *p < 0.05 vs. the control group; ^&^ *p* < 0.05 vs. UA group.

To further investigate the mechanism of CCN1 in UA-induced HK-2 cells, we conducted a series of *in vitro* experiments. As a result, HK-2 cells treated with UA showed significant NLRP3 inflammasome activation and apoptosis as well as upregulation of CCN1, which was consistent with animal experiments (Figure 4A). Besides, overexpressed CCN1 could not induce NLRP3 inflammasome activation in that the mRNA and protein expression levels of NLRP3, ASC, and pro-caspase-1 showed no remarkable change compared with the control group (Figures 4B–I). Similarly, overexpression of CCN1 could not induce cell apoptosis for, and the apoptosis-related protein expressions were not altered compared with the control group (Figures 4K–N). However, knockdown of CCN1 with short-hairpin RNA (shRNA) increased activation of the NLRP3 inflammasome(Figures 4A,B), and expression of proteins related to apoptosis increased as well (Figures 4J–N), suggesting that CCN1 played an important role in maintaining the homeostasis of cells. More importantly, CCN1 overexpression diminished UA-induced overproduction of inflammasome proteins and cytokines (*p* < 0.05, Figures 4A–N), while CCN1 silencing showed a completely opposite impact on UA-induced cell injury (*p* < 0.05, Figures 4A–N). Next, flow cytometric analysis was performed to confirm the regulation of CCN1 on cell apoptosis in HK-2, and the results were consisted with those of the above (Figure 5).

### Scutellarin Alleviated Uric Acid–Induced HK-2 Injury *via* CCN1 Regulating NLRP3

**FIGURE 6:**
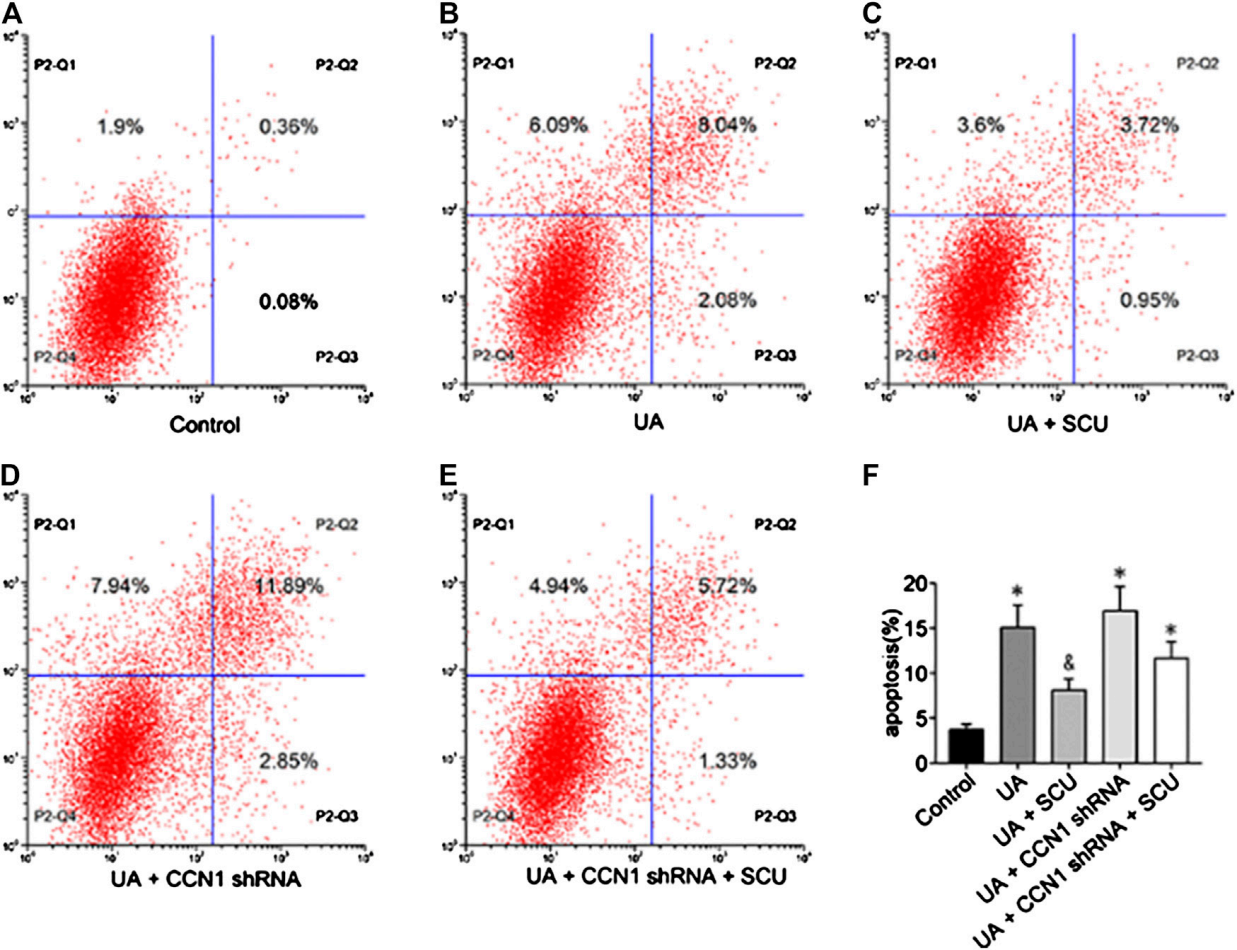
Effects of scutellarin on HK-2 cell apoptosis. Flow cytometric dot plots for HK-2 cell treated with a different manner **(A–E)**.Quantification of flow cytometry provided apoptotic rates **(F)**. *p < 0.05 vs. the control group; ^&^ *p* < 0.05 vs. UA group.

**FIGURE 7:**
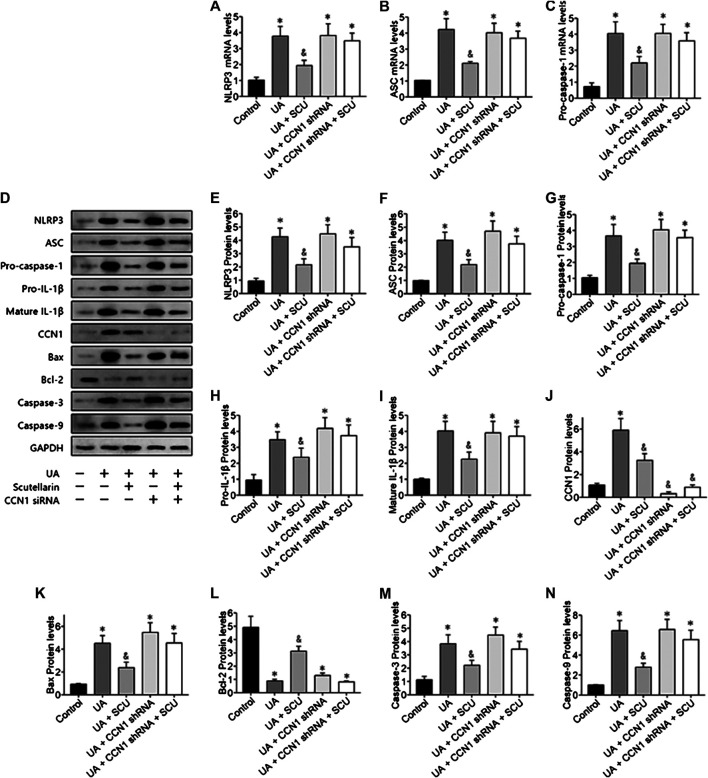
Scutellarin alleviated UA-induced HK-2 injury *via* CCN1 regulation on NLRP3. Western blot showed scutellarin significantly inhibited NLRP3 inflammasome components **(A–G)** and apoptosis-related proteins **(H–N)**, but this effect was abrogated by CCN1 shRNA. *p < 0.05 vs. the control group; ^&^ *p* < 0.05 vs. HN group.

Next, we focused on whether scutellarin could alleviate UA-induced HK-2 injury. As a result, scutellarin as a protective drug could alleviate UA-induced HK-2 injury *via* reducing cell apoptosis (Figures 6), 7J–N) and inhibiting NLRP3 inflammasome activation (Figures 7A–I). These data were in accordance with our *in vivo* results, thus providing further evidence that scutellarin could protect the kidney tubular cells from UA injury. Since both scutellarin and CCN1 played a positive role in restraining cell apoptosis and NLRP3 inflammasome activation, we further studied whether scutellarin alleviated UA-induced HK-2 injury *via* CCN1-regulating NLRP3. Intriguingly, knockdown of CCN1 not only aggravated NLRP3 inflammasome activation and apoptosis but also abrogated the protective effect of scutellarin in UA-induced HK-2 injury (Figures 6, 7). Thus, we suggested that scutellarin might inhibit HN progression by a mechanism involved in CCN1 regulating on NLRP3 inflammasome and apoptosis.

## Discussion

Scutellarin possesses strong anti-inflammatory activities in many disease (Wang W. et al., 2016; Zhao et al., 2016; Liu et al., 2017) but remains elusive whether this agent had any potential effects on HN, which, however, had been involved in various inflammatory conditions ranging from NLRP3 inflammasome activation to apoptosis. Our study confirmed that scutellarin significantly inhibited NLRP3 inflammasome activation and apoptosis in mice and HK-2 cells induced by UA, thus unraveling a previously unappreciated action mechanism for scutellarin in preventing NLRP3 inflammasome activation and apoptosis in UA-induced kidney tissues and renal tubular epithelial cell injury.

Hyperuricemia has been considered as an independent risk factor for CKD, which promotes the development and progression of CKD (Garofalo et al., 2018). Many studies suggest that a high level of serum UA is closely related to inflammatory response and renal dysfunction (Hu et al., 2009; Yang et al., 2015; Wang M.-X. et al., 2016). Gouty nephropathy caused by long-term increased UA in serum leads to the deposition of urate crystals in the kidney. UA leads to the obstruction caused by monosodium urate as well as the inflammatory response initiated by monosodium urate crystals. In fact, UA increases the levels of IL-1β, IL-6, and TNF-α in the kidney of HN mice, which leads to renal dysfunction. Moreover, MCP-1 production increases in UA-exposed HK-2 cells, which is a positive correlation with kidney injury (Wang M.-X. et al., 2016). In our study, the HN mice showed a significant decrease in renal function with the increased Scr, BUN, and kidney injury biomarkers (NGAL, cystatin C Kim-1, and IL-18). Moreover, we discovered that NLRP3 inflammasome activation in HN injury, and upregulation of Bax, cleaved caspase-3 and caspase-9, while downregulation of Bcl-2 were observed in the HN kidney tissues, which was consistent with previous studies (Wu et al., 2017; Wang et al., 2018; Tan et al., 2019; Zhang et al., 2019). Therefore, using a properly scientific approach to test whether a therapeutic drug could inhibit NLRP3 inflammasome activation and apoptosis in HN provided evidence-based guidance for clinical practice.

In the present study, scutellarin can protect renal function and mitigate tubular damage in mice with HN. Scutellarin as a natural flavone has been confirmed to have therapeutic effects against oxidative damage in many diseases. Wang Z. et al. (2016) pointed out that scutellarin protected cardiomyocytes from ischemia-reperfusion injury by reducing oxidative stress and apoptosis. Besides, scutellarin alleviates diosbulbin B-induced liver injury by inhibiting NF-κB-mediated hepatic oxidative stress and inflammation (Niu et al., 2015). In clinical practice, scutellarin has showed therapeutic effects in cerebral injury, which is related to the increase of cellular antioxidant defense capacity (Guo et al., 2011). Importantly, scutellarin was minimally toxic or nontoxic, with the maximal tolerated dose greater than 10 g/kg in mice; thus, the acute lethal dose (LD50) cannot be determined in experiment (Li et al., 2011); however, the protection effects of scutellarin in renal function of HN mice remain elusive. In the current study, we found scutellarin dose dependently alleviated the renal injury of HN model and decreased the protein expression of NGAL, Kim-1, cystatin C, and IL-18 in HN mice. Histological analysis also confirmed that scutellarin alleviated tubular damage of HN mice with a significant decline quantitative tubular injury score. Importantly, pretreatment with scutellarin inhibited NLRP3 inflammasome activation and apoptosis in HN, suggesting that its protective effect was associated with the NLRP3 and apoptosis signaling pathways.

Researchers have demonstrated that the NLRP3 inflammasome activation, which can be activated by UA crystals, is the central part in various pathological inflammatory conditions (Zhou et al., 2011; Heneka et al., 2013; Zhao et al., 2013). Innate immune function was regulated by the NLRP3 inflammasome *via* modulating the secretion and of maturation pro-inflammatory cytokines. Studies confirmed that UA improves IL-1β production in a NLRP3-dependent manner and is correlated with the HN injury (Braga et al., 2017). Zhang et al. (2019) found that in the gouty nephropathy, levels of IL-1β and IL-18 in the plasma and expression of the NLRP3 inflammasome in peripheral blood mononuclear cells were significantly increased, suggesting that the NLRP3 inflammasome played an essential role in gouty nephropathy. In this study, we found the mRNA and protein expression levels of NLRP3, ASC, and pro-caspase-1 were unregulated in the HN kidneys issues. Both pro- and mature IL-1β proteins in the kidneys were increased in HN. Pretreatment with scutellarin significantly decreased the activation of NLRP3 inflammasome and apoptosis, indicating that its protective effect was associated with NLRP3 and apoptosis. Wang Z. et al. (2016) found that the formation of ASC and oligomerization upon NLRP3 activation by ATP or nigericin can be significantly suppressed by scutellarin, implying their interference of NLRP3’s ability in recruiting ASC to inflammasome formation (Liu et al., 2017). Besides, scutellarin inhibited activation and pyroptosis of caspase-1 as well as blocking secretion of mature IL-1β into culture supernatants.

More importantly, we found an increase in CCN1 protein expression in HN kidney tissues and a decreased expression of CCN1 in HN mice administrated with scutellarin. Thus, we suggested that scutellarin alleviated HN-induced kidney injury *via* CCN1 regulation on NLRP3. Considerable studies revealed that CCN1 is an immediate early gene product pertaining to the CCN family (CCN1/Ctgf/Nov family) and also a vascular regulatory factor of extracellular matrix cross-linking (Kleer, 2016; Wu et al., 2016; Yeger and Perbal, 2016). Intriguingly, CCN1 is a key protein in the transition from acute kidney injury (AKI) to CKD, and blockade of CCN1 can mitigate renal inflammation and fibrosis after ischemic reperfusion induced AKI (Lai et al., 2014; Li et al., 2019). Our previous study (Li et al., 2018) showed that as a hub gene, CCN1 overexpressed in CKD based on 373 CKD patient samples. Second, the expression of CCN1 was positively correlated with renal fibrosis after AKI indicating that CCN1 was highly related to kidney fibrosis in a long period of time injury (Liu et al., 2017). We found that overexpressed CCN1 could not induce the activation of NLRP3 inflammasome in HK-2 cells, but HK-2 cells showed a significant NLRP3 inflammasome activation and apoptosis with CCN1 knockdown. CCN1 overexpression inhibited UA-induced overproduction of inflammasome proteins and cytokines. Knockdown of CCN1 not only aggravated NLRP3 inflammasome activation and apoptosis but also abrogated the protective effect of scutellarin in UA-induced HK-2 injury. Thus, we suggested that scutellarin might mitigate HN progression through a mechanism involved in CCN1 regulating NLRP3 inflammasome and apoptosis.

In conclusion, this study demonstrated that 1) scutellarin protected renal function and decreased SUA of HN mice, 2) protective effect of scutellarin was associated with NLRP3 and CCN1, 3) over-expressed CCN1 improved HK-2 injury induced by UA *via* negative regulation of NLRP3, and 4) scutellarin alleviated HK-2 injury induced by UA *via* CCN1 regulating NLRP3. Therefore, there is a strong rationale for the therapeutic usage of scutellarin in the treatment of HN. Furthermore, whether the anti-apoptosis and anti-NLRP3 inflammasome efficacy of scutellarin makes a difference in clinical practice requires more research to confirm for wider application.

## Data Availability Statement

The raw data supporting the conclusions of this article will be made available by the authors, without undue reservation.

## Ethics Statement

The animal study was reviewed and approved by the Ethics Committee for experimental research of the Affiliated Hospital of Qingdao University (Qingdao, China).

## Author Contributions

GL, CG, and LX are principal coinvestigators who contributed equally to study design, implementation, data analysis, and interpretation and drafting of the manuscript. LW, CY, and LZ are coinvestigators who performed the animal and cell experiments. BZ, CL, HL, and WJ are independent members who reviewed the data and took part in discussions around the observed outcomes, manuscript development, and modification. CY Li took major partinrevision, manuscript development, and language modification. YX is a coinvestigator, the senior author of this manuscript, and contributed to study design, implementation, data analysis, interpretation, manuscript development, and modification. All authors contributed to manuscript revision, reading, and approved the submitted version.

## Funding

This work was supported by the National Natural Science Foundation of China (81470973, 81770679, 81970582) and Qingdao Municipal Science and Technology Bureau (20-3-4-36-nsh). No funding bodies had any role in study design, data collection and analysis, decision to publish, or preparation of the manuscript.

## Conflict of Interest

The authors declare that the research was conducted in the absence of any commercial or financial relationships that could be construed as a potential conflict of interest.
